# Harms in Systematic Reviews Paper 3: Given the same data sources, systematic reviews of gabapentin have different results for harms

**DOI:** 10.1016/j.jclinepi.2021.10.025

**Published:** 2021-11-03

**Authors:** Riaz Qureshi, Evan Mayo-Wilson, Thanitsara Rittiphairoj, Mara McAdams-DeMarco, Eliseo Guallar, Tianjing Li

**Affiliations:** a Department of Epidemiology, Johns Hopkins Bloomberg School of Public Health, Baltimore, MD, USA; b Department of Epidemiology and Biostatistics, Indiana University School of Public Health, Bloomington, ID, USA; c Cochrane Eyes and Vision United States, University of Colorado Anschutz Medical Campus, Aurora, CO, USA; d Department of Surgery, Department of Epidemiology, Johns Hopkins School of Medicine and School of Public Health, Baltimore, MD, USA; e Department of Epidemiology, Johns Hopkins Bloomberg School of Public Health, Baltimore, MD, USA; f Department of Ophthalmology, University of Colorado Anschutz Medical Campus, Aurora, CO, USA

**Keywords:** Harms, Systematic Reviews, Meta-analysis, Synthesis, Clinical Trials

## Abstract

**Objective:**

In this methodologic study (Part 2 of 2), we examined the overlap in sources of evidence and the corresponding results for harms in systematic reviews for gabapentin.

**Study Design & Setting:**

We extracted all citations referenced as sources of evidence for harms of gabapentin from 70 systematic reviews, as well as the harms assessed and numerical results. We assessed consistency of harms between pairs of reviews with a high degree of overlap in sources of evidence (*>*50%) as determined by corrected covered area (CCA).

**Results:**

We found 514 reports cited across 70 included reviews. Most reports (244/514, 48%) were not cited in more than one review. Among 18 pairs of reviews, we found reviews had differences in which harms were assessed and their choice to meta-analyze estimates or present descriptive summaries. When a specific harm was meta-analyzed in a pair of reviews, we found similar effect estimates.

**Conclusion:**

Differences in harms results across reviews can occur because the choice of harms is driven by reviewer preferences, rather than standardized approaches to selecting harms for assessment. A paradigm shift is needed in the current approach to synthesizing harms.

## 1. Background

The current paradigm for conducting systematic reviews of interventions recommends assessing harm so that there can be a balanced discussion of potential benefits and harms; however, harms assessment is rarely the primary objective of systematic reviews [1,2]. Similarly, most randomized controlled trials are conducted to evaluate potential benefits of interventions, which they assess systematically for all participants following planned methods and using specific measurement tools or instruments (Box). By contrast, harms are often collected non-systematically; that is, harms are typically assessed through open-ended questions or spontaneous reporting by participants (Box) [3–5].

In both primary studies and systematic reviews, hundreds of harms may be observed, especially non-systematically collected harms [3–5]. Consequently, authors often use of selection criteria for reporting harms in journal articles and other reports. Selection criteria are the rules that dictate which of the identified harms are reported, usually determined by cut-offs such as the frequency of occurrence or difference between groups (e.g., “≥ 5% of participants in the intervention group”) (Box) [3–5].

Other challenges relate to the approach to seeking out evidence of harms and the choice of harms to assess. Depending on the research question, there may be important harms associated with an intervention such that reviewers *pre-specify* their interest and search the literature for relevant data to assess those harms (Box) [1]. Alternatively, reviewers might assess any harms that are identified in the literature and not pre-specify any (Box) [1]. If none are prespecified, then reviewers must choose which harms to assess and how to group related terms. Although reviewers must also decide how to group different measures of potential benefits [6–8], which are often grouped by “domain,” they must make different choices about potential harms [9]. First, reviewers must decide how to handle different words that might refer to the same type of event. Second, reviewers must decide whether and how to combine events that are similar or physiologically related. Lastly, reviewers must decide whether to undertake a general assessment of harms such as “occurrence of any harm,” and whether they will include proxies such as “loss to follow-up due to harm” (Box). Paper 1 of this series provides an overview of challenges pertaining to harms.

Systematic reviews should include all relevant reports (e.g., design papers, primary and secondary results papers, conference abstracts, trial registration) for included studies because different reports might present different and complementary information [6,7,10,11]. For overviews and studies that include systematic reviews, it is important to assess the overlap in citations so that supporting evidence is not double-counted towards a summary effect estimate [12–16].

Across a set of reviews for an intervention, we would hope to see similar results for harms, especially if those reviews include the same sources of evidence. Our objective in this paper is to evaluate whether there are differences in results for harms across reviews that include similar sources of evidence in the choices of harms to assess and the methods that lead to different effect estimates for the same harms.

## 2. Methods

The detailed methods can be found in Paper 2 of this series [17]. In brief, we searched four bibliographic databases from 1990 until September 17, 2020 with no language restrictions. Two reviewers independently screened all records independently and resolved all discrepancies through discussion. To be included in our study, we required that reviews: (i) be systematic reviews or meta-analyses; (ii) examine gabapentin for one of its commonly prescribed conditions, either on- or off-label; (iii) have any results for harms, which could have included a general statement that no harms were reported in the included studies; and (iv) be reliable in methods (i.e., a minimum set of methodologic criteria) [17]. Reliable reviews provide the “best case scenario” because they have features such are prespecified inclusion criteria and highly sensitive literature searches that might tend to produce consistent choices of harms to assess and consistent results for those harms.

For this paper, we extracted the health condition studied; whether reviews pre-specified harms for assessment; the included sources of evidence; the types of harms assessed; and the corresponding results for all reported gabapentin harms. The “results” included whether harms were assessed descriptively (i.e., presented narratively with general trends of occurrence or as multiple estimates of effect from included sources without meta-analyses) or quantitatively, with meta-analysis (Box), and any summary estimates for those which were quantitatively assessed.

### 2.1. Assessing overlap of reports included as sources of evidence

We extracted all citations that were referenced as sources of evidence for harms of gabapentin. Primary studies cited in our sample of reviews can have multiple reports. Our analyses of overlap across reviews is based on the cited reports, not the cited studies. We focused on cited reports because, for the purpose of this investigation, we considered reports for a study to be the best representation of the evidence being used across reviews. Reports from a given study often contain different information, so if two reviews include different reports from the same study, then it would not be surprising if they include different harms and associated results [6,7].

We used corrected covered area (CCA) as a tool to assess the overlap in the sources among reviews and to guide our assessment of review results for harms [18]. CAA is a citation matrix that provides a percentage of overlap in the primary sources between reviews. We calculated CCA across all reviews and by condition—defined by ourselves based on the review population—as well as for all pairwise combinations of reviews.

### 2.2. Mapping harms to standardized language

In regulatory sciences, non-systematically assessed harms are mapped to standardized terminology before they are analyzed. That is, harms are collected as many variations of what could be considered a single type of event (i.e., the “preferred term” used to enter the harm in a database) [9]. For example, “drowsiness,” “lethargy,” “sedation,” and “somnolence” are all different ways of referring to the preferred term “somnolence.” [19] A preferred term (Box) is the standardized way of referring to a specific harm and in a hierarchical system of classifying harms, such as the now arcane Coding Symbols for a Thesaurus of Adverse Reaction Terms (COSTART) (Box) or the currently used Medical Dictionary for Regulatory Activities (MedDRA) (Box) [20,21]. A preferred term is the lowest level at which analyses of harms should be conducted [9,22,23].

To standardize harms for comparison, we mapped the various ways the same event was described across reviews to the preferred terms of MedDRA. We performed our mapping by searching for each unique harm that we extracted from our reviews in the BioPortal MedDRA “Classes” (bioportal.bioontology.org/ontologies/MEDDRA?p=summary) dictionary and assigning the corresponding preferred term. All mapping of harms was performed by one investigator (RQ).

### 2.3. Analysis and synthesis

We tabulated the overlap in sources and assessed whether the corresponding results for harms across reviews differed across reviews with similar sources. We compared the harms and associated results in all pairs of reviews with a CCA of at least 50%: an amount of overlap that should be considered very high [18]. Our assessment of CCA is overall and by condition and does not account for the time of review publication; consequently, reviews conducted at different times might be more dissimilar than reviews conducted at similar times because different studies are available to include at any given point in time.

We considered differences firstly in terms of the types of harms that were reported in each review and secondly in terms of effect estimates. We considered reviews to have different results for harms if they reported different types of harms or if they reported meaningfully different effect estimates for harms that were common between reviews. If reviews used different measures (e.g., Odds Ratio, Risk Ratio, Risk Difference, Number Needed to Harm), we did not consider these as different results if the converted estimates were similar (e.g., 1/RD *≈* NNH).

## 3. Results

### 3.1. Overlap of included reports across reviews

The 70 reliable systematic reviews of gabapentin that we analyzed were published between 2001 and 2020. They cited 514 unique reports, which were published between 1990 and 2018. The number of gabapentin reports cited in a single review ranged from 1 to 161, with a median (IQR) of 6 (3 to 16). Most of the reports were cited in only a single review (244/514, 48%). The proportions cited in 2, 3, 4, or 5 reviews were 21% (107/514), 7% (38/514), 8% (39/514), and 5% (26/514), respectively. Fifty-eight (11%) reports were cited between 6 to 9 times. Two reports describing the pivotal trials submitted to the US Food and Drug Administration (US FDA) to extend marketing approval to include pain were cited in 11 and 12 reviews. APPENDIX A includes all reviews and their associated references for gabapentin by condition. Post-operative pain and neuropathic pain were the two conditions that had the greatest number of unique reports across all reviews combined, with 248 and 101 unique reports, respectively, cited across the 18 reviews for each condition (Table 1). The lowest numbers of gabapentin reports appeared in the single review of restless leg syndrome and the single review of psychiatric disorders, which included 2 and 1 citations (Table 1).

Overall, the CCA was low at 2%. The CCA varied widely when calculated by condition, with epilepsy having the lowest of 3% (12 reviews with 84 citations of 63 unique reports) and alcohol dependence having the highest at 21% (3 reviews with 20 citations of 14 unique reports) (Table 1). APPENDIX B contains further exploration of the overlap in sources of evidence between conditions.

### 3.2. Harms of gabapentin

**Fig. 1.**
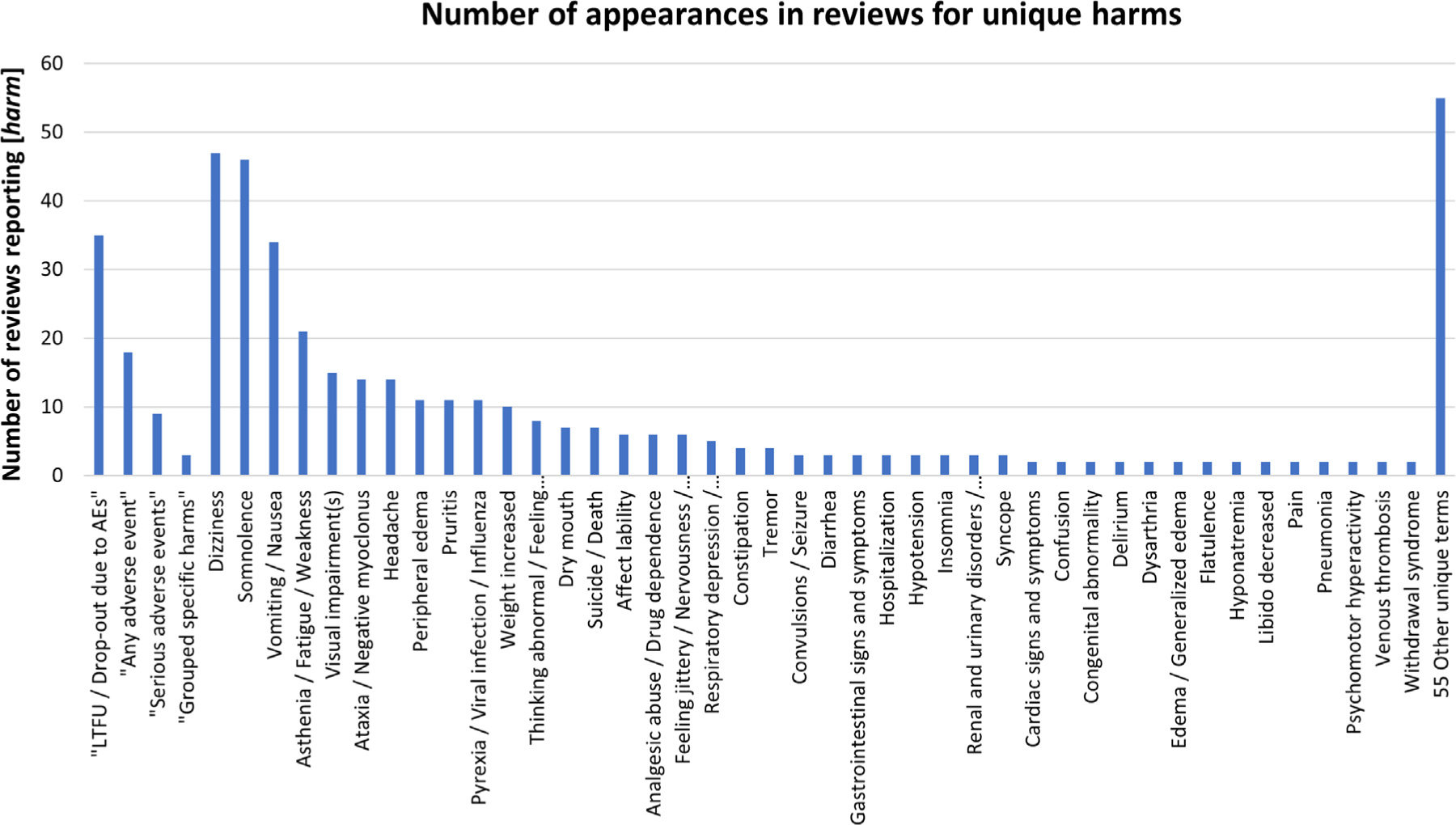
Number of appearances in reviews for unique harms.

Across the 70 reviews, we identified 167 reported gabapentin harms before mapping to MedDRA preferred terms. After we mapped the terms, we found reviewers assessed 97 specific harms (e.g., Dizziness, Somnolence, Vomiting/Nausea). Reviewers also used three general methods of assessing harms—“any non-specific harm”, “serious adverse events”, and “grouped specific harms” (i.e., a composite of multiple harms). Reviewers also assessed a proxy for harm, “loss to follow up or drop out due to harms”. Most reviews used a general or proxy method in addition to assessing specific harms. No reviews assessed harms at a higher order category such as mid-level Nervous system harms; however, the general “grouped specific harms” sometimes assessed harms under a single higher order category (e.g., occurrence of dizziness, staggering, unsteadiness, or vertigo). Fig. 1 presents the number of times each mapped harm was assessed across the 70 reviews. Most of the specific harms did not appear in more than one review (55/97, 57%); the ten most commonly reported harms were: Dizziness, Somnolence, Vomiting/Nausea, Asthenia/Fatigue/Weakness, Visual impairment(s), Ataxia/Negative myoclonus, Headache, Peripheral edema, Pruritis, and Pyrexia/Viral infection/Influenza (Fig. 1).

Of the 97 specific harms, 78 (80%) were only ever descriptively assessed and 19 (20%) were quantitatively assessed in one or more reviews–APPENDIX C contains the estimates of effect from these meta-analyses. Estimates tended to be non-significant. Harms with statistically significant associations with gabapentin included Dizziness, Somnolence, Ataxia/Negative myoclonus, Peripheral edema, Visual disturbances, and Mentation/Abnormal thinking. Some reviews reported statistically significant protective effects for Vomiting/Nausea. APPENDIX D contains the 167 unique harms that were reported for gabapentin across the 70 included reviews and the 97 corresponding mapped MedDRA preferred terms.

Of 2415 pairwise comparisons between reviews, we found 18 pairs of reviews with more than 50% overlap in the reports cited for gabapentin (Table 2). As expected, where estimates of effect were presented for the same harm, most pairs of reviews had similar results. However, there were large differences in the specific harms reported among pairs of reviews with high overlap. These differences arose because of the reviewers’ chosen selection criteria for reporting harms and their approach to assessing harm. For example, in a pair with 100% overlap in a given set of included primary studies, one review may choose to describe a larger set of harms in a descriptive way while the other review focuses only on one or a few quantitative assessments (Table 2).

## 4. Discussion

Systematic reviewers already face many challenges in synthesizing harms, including: multiple types of evidence required in addition to randomized controlled trials; the collection of harms in primary studies is not standardized; harms are often underreported in primary studies; and analysis of harms is difficult, even when given full participant data [9]. While guidelines exist for reviewers to address some of these challenges, we are unaware of any other studies examining overlap and results for harms across systematic reviews. In this study, we uncovered two additional obstacles to the reliability of review conclusions for harms: the choice of harms for assessment and the use of non-standardized language to refer to harms.

When we examined the overlapping reports and results for harms across systematic reviews of gabapentin, we discovered that reviews often differed in the choice of harms to assess and the approach for analyzing harms. We did not find evidence of prespecified rationales, or of consistent patterns, for choosing which harms to assess, which suggests that harms may be selected based on reviewers’ preferences. For example, we found that even when two reviews cited the exact same included reports as sources of evidence, the types of harms and the approach taken to assess them could be very different. When pairs of reviews with high overlap reported the same harms with meta-analytic effect estimates, the estimates were often similar when considering direction and magnitude. However, when the same harms appeared in multiple reviews, there were discrepancies when considering the decision to pool estimates into a summary effect or to present a descriptive summary. Additionally, across meta-analyses from the broader sample of reviews (APPENDIX C) there were differences in statistical significance and the subsequent conclusions made about potential harms.

Our expectation that reviews would have similar results for harms if they included similar sources of evidence was met only when the same harms were assessed using the same approach (e.g., meta-analyzed across included studies). In the absence of core outcome sets for harms, and lacking any strong community norms, reviewers have considerable freedom to choose their approach to assessing harms (i.e., pre-specification of harms vs. not pre-specifying any harms) and to apply their own selection criteria in deciding which harms to assess and report. This freedom can lead to important differences across reviews, even when they cite the same evidence: authors of one review may decide to assess harm using a single proxy such as “drop out due to harms”, whereas authors of another review may choose to assess and report all specific harms identified in the included studies. It is a common practice to limit the number of outcomes assessed in a review and to include “harms” as a single outcome by trying to summarize and create a composite for harm, particularly in when following the Grading of Recommendations Assessment, Development and Evaluation approach and creating a Summary of findings table [24]. The challenges with this practice are that there exists no standardized way to do this and limiting the number of harms assessed to some small number imposes prioritization that may or may not be appropriate. The process of selecting harms to synthesize could become more standardized to improve consistency, for example assessing harms that patients consider most important [25]. Of course, different patients might consider the same harm as more or less important, so limiting reviews to certain harms might limit their generalizability. Moreover, too much pre-specification might limit the ability of systematic reviews to discover evidence of harms and contribute to understanding new associations over time. The potential for differences in harms across reviews should be considered when conducting an overview of reviews and by evidence users—from patients to clinicians and guideline developers: not all reviews for a given clinical question will provide the same information about harms because the methods used to assess harms are not actually systematic.

Although standardized systems for describing harms such as MedDRA have existed for decades and are used in regulatory research, systematic reviewers often use common, non-standardized, language to refer to harms. This use of non-standardized language means that the same harms are described to using different terms between reviews; for example, multiple reviews assessing the risks of “Drowsiness,” “Lethargy,” “Sedation,” or “Somnolence” when these all describe the standardized preferred term “Somnolence”. This creates a major challenge for evidence users. When primary studies use common terminology to refer to harms, reviewers could standardize language and terms (e.g., if Trial A reports “Drowsiness” and Trial B reports “Lethargy”, then the reviewers could code both as “Somnolence”). Standardized systems are also hierarchical in nature, which provides appropriate ways to aggregate harms using higher order terms. For example, if reviewers mapped specific harms of interest—pre-specified or otherwise—to corresponding mid-level systems (e.g., nervous system) and conducted analyses at the mid-level, then they could draw broader conclusions about the types of harms that patient might expect. Combining related harms using these systems increases statistical power to detect effects, and existing systems might be more appropriate and more easily comparable than *ad hoc* composites created by reviewers.

Lastly, systematic reviewers should state their rationales for pre-specifying harms to include, or for not prespecifying harms to include, and their reasons for choosing harms for reporting. If reviewers explain their choice of approach and selection criteria, then readers will be better able to contextualize the results. Better reporting of reviewer decisions and review limitations could reduce the likelihood that conclusions are overinterpreted.

## 5. Conclusion

We found that among systematic reviews of gabapentin, reviews that took the same approach to assessing the same harms found similar effect estimates; however, reviews often assessed different harms, and reviews often used different methods to assess harms (i.e., descriptive or quantitative). Trialists and systematic reviewers should use standardized language when referring to harms so that harms will be more consistently described across reviews. Reviewers should explain the rationale for selecting harms to assess and report. Readers should be aware that conclusions about harms may be unreliable; the types of harms and conclusions about harms in a systematic review might differ from other reviews of the same drug and health condition, even when both reviews include the same sources of evidence.

## Supplementary Material

1

2

3

4

## Figures and Tables

**Table 1.** Review populations and overlap of primary reports among reviews

Review population^i^	Number of reviews (R)	Number of citation appearances (C)	Number of unique cited sources (U)	Corrected Covered Area^ii^
Neuropathic pain	18	213	101	7%
Epilepsy	12	84	63	3%
Vasomotor symptoms	4	26	18	15%
Postherpetic neuralgia	7	59	34	12%
Post-operative pain	18	780	248	13%
Restless leg syndrome	1	2	2	NA
Migraine	4	17	12	14%
Fibromyalgia	6	49	38	6%
Alcohol dependence	3	20	14	21%
Psychiatric disorders	1	1	1	NA
Non-specific	7	119	84	7%

i Reviews could include multiple conditions/populations

ii Corrected Covered Area = (C – U) / ((U ∗ R) – U)

**Table 2.** Gabapentin harms reported in pairs of reviews with Corrected Covered Area (CCA) *≥* 50% (n=18 pairs of reviews)

Harms reported	Summary effect estimate^i^	# trials^ii^	Harms reported	Summary effect estimate^i^	# trials^ii^
Fabritius 2017a (Post-operative Pain; n = 75 sources) versus Fabritius 2017b (Post-operative Pain; n = 122 sources)					52% CCA
Dizziness	RR = 1.00 (0.88, 1,12)	37	Dizziness	RR = 1.06 (0.94, 1,21)	52
Nausea	RR = 0.81 (0.72, 0.92)	33	Nausea	RR = 0.81 (0.72, 0.91)	49
Sedation	RR = 1.50 (1.13, 1.99)	31	Sedation	RR = 1.32 (1.07, 1.65)	42
“Serious AEs”	RR = 1.12 (0.71, 1.77)	15	“Serious AEs”	OR = 1.22 (0.72, 2.06)	27
Vomiting	RR = 0.75 (0.63, 0.89)	29	Vomiting	RR = 0.79 (0.67, 0.92)	44
			Admission to ICU	Reported occurrence or trend^iii^	NA
			Infection	Reported occurrence or trend	NA
			Pneumonia	Reported occurrence or trend	NA
			Prolonged hospital stay	Reported occurrence or trend	NA
			Suicide/death	Reported occurrence or trend	NA
			Vein thrombosis	Reported occurrence or trend	NA
Fabritius 2016 (Post-operative Pain; n = 135 sources) versus Fabritius 2017a (Post-operative Pain; n = 75 sources)					54%CCA
Dizziness	RR = 1.02 (0.9, 1.1)	60	Dizziness	RR = 1.00 (0.88, 1,12)	37
Nausea	RR = 0.82 (0.7, 0.9)	57	Nausea	RR = 0.81 (0.72, 0.92)	33
Sedation	RR = 1.33 (1.0, 1.3)	51	Sedation	RR = 1.50 (1.13, 1.99)	31
“Serious AEs”	RR = 1.14 (0.6, 2.1)	26	“Serious AEs”	RR = 1.12 (0.71, 1.77)	15
Vomiting	RR = 0.80 (0.7, 0.9)	51	Vomiting	RR = 0.75 (0.63, 0.89)	29
Atrial fibrillation	Reported occurrence or trend	NA			
Atelactasis	Reported occurrence or trend	NA			
Feeling jittery	Reported occurrence or trend	NA			
Hospital readmission	Reported occurrence or trend	NA			
Major bleed	Reported occurrence or trend	NA			
Numb fingers/tongue/mouth	Reported occurrence or trend	NA			
Pneumonia	Reported occurrence or trend	NA			
Pleura effusion	Reported occurrence or trend	NA			
Pruritis	Reported occurrence or trend	NA			
Respiratory arrest	Reported occurrence or trend	NA			
Suicide/death	Reported occurrence or trend	NA			
Urinary retention	Reported occurrence or trend	NA			
Vein thrombosis	Reported occurrence or trend	NA			
Selph 2011 (Neuropathic Pain; n = 21 sources) versus Moore 2014 (Neuropathic Pain & Fibromyalgia; n = 29 sources)					56% CCA
Ataxia/Negative myoclonus	No estimate for gabapentin alone ^iv^	NA	Ataxia/Negative myoclonus	RR = 4.5 (1.9, 11)	5
Dizziness	Reported occurrence or trend	NA	Dizziness	RR = 3.1 (2.6, 3.8)	21
“LTFU due to AEs”	Multiple estimates in review^v^	NA	“LTFU due to AEs”	RR = 1.4 (1.1, 1.7)	22
			“Any AE”	RR = 1.25 (1.2, 1.3)	17
Blurred vision	RR = 1.56 (0.12, 20.97)	2			
Dry Mouth	No estimate for gabapentin alone	NA			
					
			Peripheral edema	RR = 3.3 (2.2, 4.9)	12
			“Serious AEs”	RR = 1.2 (0.8, 1.7)	19
			Somnolence	RR = 2.9 (2.3, 2.6)	20
			Suicide/death	Multiple estimates in review	NA
Finnerup 2010 (Neuropathic Pain & Postherpetic Neuralgia; n = 15 sources) versus Selph 2011 (Neuropathic Pain; n = 21 sources)					57% CCA
“LTFU due to AEs”	NNH = 32.5 (18, 122)	14	“LTFU due to AEs”	Multiple estimates in review	NA
			Ataxia/Negative myoclonus	No estimate for gabapentin alone	NA
					
			Blurred vision	RR = 1.56 (0.12, 20.97)	2
			Dizziness	Reported occurrence or trend	NA
			Dry mouth	No estimate for gabapentin alone	NA
Wareham 2007 (Postherpetic Neuralgia; n = 6 sources) versus Watson 2010 (Postherpetic Neuralgia; n = 5 sources)					57% CCA
Ataxia/negative myoclonus	Reported occurrence or trend	NA	Ataxia/negative myoclonus	Reported occurrence or trend	NA
Dizziness	Reported occurrence or trend	NA	Dizziness	Reported occurrence or trend	NA
Infection	Reported occurrence or trend	NA	Infection	Reported occurrence or trend	NA
“LTFU due to AEs”	Reported occurrence or trend	NA	“LTFU due to AEs”	Reported occurrence or trend	NA
Peripheral edema	Reported occurrence or trend	NA	Peripheral edema	Reported occurrence or trend	NA
Somnolence	Reported occurrence or trend	NA	Somnolence	Reported occurrence or trend	NA
Rudroju 2013 (Neuropathic Pain; n = 7 sources) versus Griebeler 2014 (Neuropathic Pain; n = 4 sources)					57% CCA
			Abdominal pain	Reported occurrence or trend	NA
			Amnesia	Reported occurrence or trend	NA
			Ataxia/negative myoclonus	Reported occurrence or trend	NA
			Blurred vision	Reported occurrence or trend	NA
			Concentration problems	Reported occurrence or trend	NA
			Constipation	Reported occurrence or trend	NA
			Diarrhea	Reported occurrence or trend	NA
			Diplopia	Reported occurrence or trend	NA
			Disarthria	Reported occurrence or trend	NA
			Dizziness	Reported occurrence or trend	NA
			Dyspepsia	Reported occurrence or trend	NA
			Emotional lability	Reported occurrence or trend	NA
			Fatigue	Reported occurrence or trend	NA
			Flatulence	Reported occurrence or trend	NA
			Headache	Reported occurrence or trend	NA
			Hostile behaviour	Reported occurrence or trend	NA
			Hyperactive behaviour	Reported occurrence or trend	NA
			Hyperkinesia	Reported occurrence or trend	NA
			Hypersensitivity reactions	Reported occurrence or trend	NA
			Incoordination	Reported occurrence or trend	NA
“LTFU due to AEs”	OR = 0.70 (0.25, 1.85)	NR			
			Mood swings	Reported occurrence or trend	NA
			Myalgia	Reported occurrence or trend	NA
			Nausea	Reported occurrence or trend	NA
			Nervousness	Reported occurrence or trend	NA
			Nystagmus	Reported occurrence or trend	NA
			Peripheral edema	Reported occurrence or trend	NA
			Restlessness	Reported occurrence or trend	NA
			Seizures	Reported occurrence or trend	NA
			Somnolence	Reported occurrence or trend	NA
			Suicidal thoughts/behaviour	Reported occurrence or trend	NA
			Tremors	Reported occurrence or trend	NA
			Viral respiratory tract infections	Reported occurrence or trend	NA
			Vomiting	Reported occurrence or trend	NA
			Weight gain	Reported occurrence or trend	NA
			Withdrawal symptoms	Reported occurrence or trend	NA
			Xerostomia	Reported occurrence or trend	NA
Doleman 2015 (Post-operative Pain; n = 133 sources) versus Fabritius 2017b (Post-operative Pain; n = 122 sources)					57% CCA
Dizziness	RR = 1.04 (0.94, 1.15)	51	Dizziness	RR = 1.06 (0.94, 1,21)	52
Nausea	RR = 0.78 (0.69, 0.87)	58	Nausea	RR = 0.81 (0.72, 0.91)	49
Sedation	RR = 1.18 (1.09, 1.28)	52	Sedation	RR = 1.32 (1.07, 1.65)	42
Vomiting	RR = 0.67 (0.59, 0.76)	57	Vomiting	RR = 0.79 (0.67, 0.92)	44
			Admission to ICU	Reported occurrence or trend	NA
Confusion	RR = 0.50 (0.19, 1.34)	3			
Constipation	RR = 0.80 (0.44, 1.44)	10			
Headache	RR = 1.05 (0.82, 1.33)	24			
			Infection	Reported occurrence or trend	NA
			Pneumonia	Reported occurrence or trend	NA
			Prolonged hospital stay	Reported occurrence or trend	NA
Pruritis	RR = 0.64 (0.51, 0.80)	29			
Respiratory depression	RR = 0.97 (0.45, 2.10)	6			
			“Serious AEs”	OR = 1.22 (0.72, 2.06)	27
			Suicide/death	Reported occurrence or trend	NA
Urinary retention	RR = 0.64 (0.40, 1.04)	14			
			Vein thrombosis	Reported occurrence or trend	NA
Visual disturbance	RR = 1.36 (0.77, 2.40)	4			
Maguire 2011 (Epilepsy & Non-specific; n = 3 sources) versus Nevitt 2017 (Epilepsy; n = 5 sources)					60% CCA
Asthenia	Reported occurrence or trend	NA	Asthenia	Reported occurrence or trend	NA
Dizziness	Reported occurrence or trend	NA	Dizziness	Reported occurrence or trend	NA
Headache	Reported occurrence or trend	NA	Headache	Reported occurrence or trend	NA
“LTFU due to AEs”	Reported occurrence or trend	NA	“LTFU due to AEs”	Reported occurrence or trend	NA
Weight gain	Reported occurrence or trend	NA	Weight gain	Reported occurrence or trend	NA
			Accidental injury	Reported occurrence or trend	NA
			Anorexia/weight loss	Reported occurrence or trend	NA
			“Any AE”	Reported occurrence or trend	NA
			Aphasia	Reported occurrence or trend	NA
			Ataxia/negative myoclonus	Reported occurrence or trend	NA
			Cognitive problems	Reported occurrence or trend	NA
			Dental problems	Reported occurrence or trend	NA
			Depression	Reported occurrence or trend	NA
			Drowsiness	Reported occurrence or trend	NA
			Fever or viral infection	Reported occurrence or trend	NA
			Gastrointestinal disturbances	Reported occurrence or trend	NA
			Hair loss	Reported occurrence or trend	NA
Hyponatremia	Reported occurrence or trend	NA			
			Impotence	Reported occurrence or trend	NA
			Increased seizures	Reported occurrence or trend	NA
			Infection	Reported occurrence or trend	NA
			Laboratory results abnormal	Reported occurrence or trend	NA
			Menstrual problems	Reported occurrence or trend	NA
			Mood or behaviour changes	Reported occurrence or trend	NA
			Nausea/vomiting	Reported occurrence or trend	NA
			Pain	Reported occurrence or trend	NA
			Paraesthesia/tingling	Reported occurrence or trend	NA
			Problems sleeping/nightmares	Reported occurrence or trend	NA
			Rash/skin disorder	Reported occurrence or trend	NA
			Renal/urinary disorder	Reported occurrence or trend	NA
			Respiratory disorder	Reported occurrence or trend	NA
			Tremor/twitch	Reported occurrence or trend	NA
			Visual disturbance/nystagmus	Reported occurrence or trend	NA
Doleman 2015 (Post-operative Pain; n = 133 sources) versus Fabritius 2016 (Post-operative Pain; n = 135 sources)					61% CCA
Dizziness	RR = 1.04 (0.94, 1.15)	51	Dizziness	RR = 1.02 (0.9, 1.1)	60
Nausea	RR = 0.78 (0.69, 0.87)	58	Nausea	RR = 0.82 (0.7, 0.9)	57
Pruritis	RR = 0.64 (0.51, 0.80)	29	Pruritis	Reported occurrence or trend	NA
Respiratory depression	RR = 0.97 (0.45, 2.10)	6	Respiratory arrest	Reported occurrence or trend	NA
Sedation	RR = 1.18 (1.09, 1.28)	52	Sedation	RR = 1.33 (1.0, 1.3)	51
Urinary retention	RR = 0.64 (0.40, 1.04)	14	Urinary retention	Reported occurrence or trend	NA
Vomiting	RR = 0.67 (0.59, 0.76)	57	Vomiting	RR = 0.80 (0.7, 0.9)	51
			Atelactasis	Reported occurrence or trend	NA
			Atrial fibrillation	Reported occurrence or trend	NA
Confusion	RR = 0.50 (0.19, 1.34)	3			
Constipation	RR = 0.80 (0.44, 1.44)	10			
			Feeling jittery	Reported occurrence or trend	NA
Headache	RR = 1.05 (0.82, 1.33)	24			
			Hospital readmission	Reported occurrence or trend	NA
			Major bleed	Reported occurrence or trend	NA
			Numb fingers/tongue/mouth	Reported occurrence or trend	NA
			Pleura effusion	Reported occurrence or trend	NA
			Pneumonia	Reported occurrence or trend	NA
			“Serious AEs”	RR = 1.14 (0.6, 2.1)	26
			Suicide/death	Reported occurrence or trend	NA
			Vein thrombosis	Reported occurrence or trend	NA
Visual disturbance	RR = 1.36 (0.77, 2.40)	4			
Moore 2014 (Neuropathic Pain & Fibromyalgia; n = 29 sources) versus Wiffen 2017 (Neuropathic Pain; n = 33 sources)					65% CCA
“Any AE”	RR = 1.25 (1.2, 1.3)	17	“Any AE”	RR = 1.28 (1.22, 1.36)	18
Ataxia/Negative myoclonus	RR = 4.5 (1.9, 11)	5	Ataxia/Negative myoclonus	RR = 5.53 (2.49, 12.28)	4
Dizziness	RR = 3.1 (2.6, 3.8)	21	Dizziness	RR = 2.87 (2.40, 3.44)	21
“LTFU due to AEs”	RR = 1.4 (1.1, 1.7)	22	“LTFU due to AEs”	RR = 1.38 (1.14, 1.67)	22
Peripheral edema	RR = 3.3 (2.2, 4.9)	12	Peripheral edema	RR = 4.12 (2.66, 6.39)	12
“Serious AEs”	RR = 1.2 (0.8, 1.7)	19	“Serious AEs”	RR = 1.19 (0.83, 1.71)	19
Somnolence	RR = 2.9 (2.3, 2.6)	20	Somnolence	RR = 2.82 (2.27, 3.50)	20
Suicide/death	Multiple estimates in review	NA			
Finnerup 2005 (Neuropathic Pain; n = 10 sources) versus Finnerup 2010 (Neuropathic Pain & Postherpetic Neuralgia; n = 15 sources)					67% CCA
“LTFU due to AEs”	NNH = 26.1 (14.1, 170)	NR	“LTFU due to AEs”	NNH = 32.5 (12, 122)	14
“Any AE”	Reported occurrence or trend	NA			
Smith 2016 (Non-specific; n = 33 sources) versus Evoy 2017 (Non-specific; n = 32 sources)					71% CCA
Death	Reported occurrence or trend	NA	Death	Reported occurrence or trend	NA
GBP Abuse/misuse	Reported occurrence or trend	NA	GBP Abuse/misuse	Reported occurrence or trend	NA
Addiction	Reported occurrence or trend	NA			
Anisocoria	Reported occurrence or trend	NA			
Ataxia/negative myoclonus	Reported occurrence or trend	NA			
Bradycardia	Reported occurrence or trend	NA			
Cardiac symptoms	Reported occurrence or trend	NA			
CNS symptoms	Reported occurrence or trend	NA			
Coma	Reported occurrence or trend	NA			
Delirium	Reported occurrence or trend	NA			
Dependency	Reported occurrence or trend	NA			
Depressed gag reflex	Reported occurrence or trend	NA			
Diarrhea	Reported occurrence or trend	NA			
Dizziness	Reported occurrence or trend	NA			
Drowsiness	Reported occurrence or trend	NA			
Dysphoria	Reported occurrence or trend	NA			
Dystonia	Reported occurrence or trend	NA			
Emotional lability	Reported occurrence or trend	NA			
Feeling “high/stoned”	Reported occurrence or trend	NA			
GI symptoms	Reported occurrence or trend	NA			
Hypotension	Reported occurrence or trend	NA			
Hypoxia	Reported occurrence or trend	NA			
Hyperreflexic	Reported occurrence or trend	NA			
Lethargy	Reported occurrence or trend	NA			
Metabolic signs	Reported occurrence or trend	NA			
Neuromuscular symptoms	Reported occurrence or trend	NA			
Nystagmus	Reported occurrence or trend	NA			
Reduced cocaine cravings	Reported occurrence or trend	NA			
Respiratory depression	Reported occurrence or trend	NA			
Slurred speech	Reported occurrence or trend	NA			
Syncope	Reported occurrence or trend	NA			
Tachycardia	Reported occurrence or trend	NA			
Tremulous	Reported occurrence or trend				
Vomiting/nausea	Reported occurrence or trend	NA			
Withdrawal	Reported occurrence or trend				
Maguire 2011 (Epilepsy & Non-specific; n = 3 sources) versus Campos 2016 (Epilepsy; n = 4 sources)					75% CCA
“LTFU due to AEs”	Reported occurrence or trend	NA	“LTFU due to AEs”	Multiple estimates in review	NA
Asthenia	Reported occurrence or trend	NA			
Dizziness	Reported occurrence or trend	NA			
Headache	Reported occurrence or trend	NA			
Hyponatremia	Reported occurrence or trend	NA			
Weight gain	Reported occurrence or trend	NA			
Shanthanna 2017 (Neuropathic Pain; n = 3 sources) versus Enke 2018 (Neuropathic Pain; n = 4 sources)					75% CCA
			“Any AE”	No estimate for gabapentin alone	NA
Constipation	Reported occurrence or trend	NA			
Dizziness	Reported occurrence or trend	NA			
Drowsiness	Reported occurrence or trend	NA			
Fatigue	Reported occurrence or trend	NA			
Forgetfulness	Reported occurrence or trend	NA			
Headache	Reported occurrence or trend	NA			
“LTFU due to AEs”	Reported occurrence or trend	NA			
Mentation	Reported occurrence or trend	NA			
Pruritis	Reported occurrence or trend	NA			
Restlessness	Reported occurrence or trend	NA			
			“Serious AEs”	No estimate for gabapentin alone	NA
Visual accommodation / blurred vision	Reported occurrence or trend	NA			
Visual disturbances	Reported occurrence or trend	NA			
Vomiting/nausea	Reported occurrence or trend	NA			
Campos 2016 (Epilepsy; n = 4 sources) versus Nevitt 2017 (Epilepsy; n = 5 sources)					80% CCA
“LTFU due to AEs”	Multiple estimates in review	NA	“LTFU due to AEs”	Multiple estimates in review	NA
			Accidental injury	Reported occurrence or trend	NA
			Anorexia/weight loss	Reported occurrence or trend	NA
			“Any AE”	Reported occurrence or trend	NA
			Aphasia	Reported occurrence or trend	NA
			Asthenia	Reported occurrence or trend	NA
			Ataxia/negative myoclonus	Reported occurrence or trend	NA
			Cognitive problems	Reported occurrence or trend	NA
			Dental problems	Reported occurrence or trend	NA
			Depression	Reported occurrence or trend	NA
			Dizziness	Reported occurrence or trend	NA
			Drowsiness	Reported occurrence or trend	NA
			Fever or viral infection	Reported occurrence or trend	NA
			Gastrointestinal disturbances	Reported occurrence or trend	NA
			Hair loss	Reported occurrence or trend	NA
			Headache	Reported occurrence or trend	NA
			Impotence	Reported occurrence or trend	NA
			Increased seizures	Reported occurrence or trend	NA
			Infection	Reported occurrence or trend	NA
			Laboratory results abnormal	Reported occurrence or trend	NA
			Menstrual problems	Reported occurrence or trend	NA
			Mood or behavior changes	Reported occurrence or trend	NA
			Nausea/vomiting	Reported occurrence or trend	NA
			Pain	Reported occurrence or trend	NA
			Paraesthesia/tingling	Reported occurrence or trend	NA
			Problems sleeping/nightmares	Reported occurrence or trend	NA
			Rash/skin disorder	Reported occurrence or trend	NA
			Renal/urinary disorder	Reported occurrence or trend	NA
			Respiratory disorder	Reported occurrence or trend	NA
			Tremor/twitch	Reported occurrence or trend	NA
			Visual disturbance/nystagmus	Reported occurrence or trend	NA
			Weight gain	Reported occurrence or trend	NA
Fabritius 2016 (Post-operative Pain; n = 135 sources) versus Fabritius 2017b (Post-operative Pain; n = 122 sources)					88% CCA
Dizziness	RR = 1.02 (0.9, 1.1)	60	Dizziness	RR = 1.06 (0.94, 1,21)	52
Nausea	RR = 0.82 (0.7, 0.9)	57	Nausea	RR = 0.81 (0.72, 0.91)	49
Pneumonia	Reported occurrence or trend	NA	Pneumonia	Reported occurrence or trend	NA
Sedation	RR = 1.33 (1.0, 1.3)	51	Sedation	RR = 1.32 (1.07, 1.65)	42
“Serious AEs”	RR = 1.14 (0.6, 2.1)	26	“Serious AEs”	OR = 1.22 (0.72, 2.06)	27
Suicide/death	Reported occurrence or trend	NA	Suicide/death	Reported occurrence or trend	NA
Vein thrombosis	Reported occurrence or trend	NA	Vein thrombosis	Reported occurrence or trend	NA
Vomiting	RR = 0.80 (0.7, 0.9)	51	Vomiting	RR = 0.79 (0.67, 0.92)	44
			Admission to ICU	Reported occurrence or trend	NA
Atelectasis	Reported occurrence or trend	NA			
Atrial fibrillation	Reported occurrence or trend	NA			
Feeling jittery	Reported occurrence or trend	NA			
Hospital readmission	Reported occurrence or trend	NA			
			Infection	Reported occurrence or trend	NA
Major bleed	Reported occurrence or trend	NA			
Numb fingers/tongue/mouth	Reported occurrence or trend	NA			
Pleura effusion	Reported occurrence or trend	NA			
			Prolonged hospital stay	Reported occurrence or trend	NA
Pruritis	Reported occurrence or trend	NA			
Respiratory arrest	Reported occurrence or trend	NA			
Urinary retention	Reported occurrence or trend	NA			
Linde 2013 (Migraine; n = 5 sources) versus Mulleners 2015 (Migraine; n = 5 sources)					100% CCA
Abnormal thinking	RD = 0.05 (0.01, 0.09)	3	Abnormal thinking	NNH = 20 (11, 100)	NR
“Any AE”	RD = 0.05 (-0.04, 0.14)	3	“Any AE”	Reported occurrence or trend	NA
Dizziness	RD = 0.15 (0.08, 0.22)	3	Dizziness	NNH = 7 (5, 13)	NR
Fatigue	RD = -0.03 (-0.08, 0.03)	3	Fatigue	Reported occurrence or trend	NA
Flu syndrome	RD = 0.03 (-0.03, 0.08)	2	Flu syndrome	NNH = 7 (4, 25)	NR
“LTFU due to AEs”	Reported occurrence or trend	NA	“LTFU due to AEs”	Reported occurrence or trend	NA
Somnolence	RD = 0.11 (0.03, 0.18)	2	Somnolence	NNH = 9 (6, 33)	NR
Asthenia	RD = -0.03 (-0.08, 0.03)	3			
Ataxia/negative myoclonus	Reported occurrence or trend	NA			
Back pain	Reported occurrence or trend	NA			
Confusion	Reported occurrence or trend	NA			
Diarrhea	Reported occurrence or trend	NA			
Dry mouth	Reported occurrence or	NA			
Flatulence	Reported occurrence or trend	NA			
Headache	Reported occurrence or trend	NA			
Infection	Reported occurrence or trend	NA			
Nausea	Reported occurrence or trend	NA			
Nervousness	Reported occurrence or trend	NA			
Pain	Reported occurrence or trend	NA			
Pharyngitis	Reported occurrence or trend	NA			
Sinusitis	Reported occurrence or trend	NA			
Tremor	Reported occurrence or trend	NA			
Üçeyler 2013 (Fibromyalgia; n = 1 source) versus Cooper 2017 (Fibromyalgia; n = 1 source)					100% CCA
Dizziness	RR = 2.71 (1.21, 6.07)	1	Dizziness	Reported occurrence or trend	NA
Lightheadedness	Reported occurrence or trend	NA	Lightheadedness	Reported occurrence or trend	NA
“LTFU due to AEs”	RR = 1.71 (0.71, 4.11)	1	“LTFU due to AEs”	Reported occurrence or trend	NA
Sedation	Reported occurrence or trend	NA	Sedation	Reported occurrence or trend	NA
			“Serious AEs”	Reported occurrence or trend	NA
Weight gain	Reported occurrence or trend	NA			

i Single summary effect estimate for “gabapentin [any dose] vs. [any comparator]”, if available, as reported in the systematic review. Multiple estimates were not extracted.

ii Number of trials included in meta-analysis for summary estimate; “Not Reported” (NR) if the number of trials contributing to meta-analysis was not reported; “Not Applicable” (NA) if no pooling was done for harm and the review qualitatively synthesized the harmCell colour: Grey = Difference between reviews in a pair, either because a harm was reported in only one review or because the two reviews reported meaningfully different effect estimatesNotes:

iii “Reported occurrence or trend” – Review reported that a harm occurred using simple counts/proportions with no effect estimate or reported a qualitative description of the harm (e.g., “Trials with low risk of bias reported the following [harms]: …” or “Gabapentin significantly increased the proportion of people who experienced …”).

iv “No estimate for gabapentin alone” – Review pooled gabapentin with other treatments in meta-analysis and did not present an estimate of gabapentin on its own vs. a comparator (e.g., gabapentin and pregabalin vs. placebo).

v “Multiple estimates in review” – Review reported either multiple estimates from included studies and did not conduct a meta-analysis of those estimates, or conducted multiple meta-analyses for gabapentin with no overall estimate (e.g., high-dose gabapentin vs. comparator, low-dose gabapentin vs. comparator; gabapentin vs. lamotrigine, gabapentin vs. carbamazepine, gabapentin vs. pregabalin).

## What is new?

- Even when systematic reviews used similar sources of evidence, we found inconsistency in the results for harms, which was attributable to the choice of harms to assess and the decisions to perform meta-analysis or summarize effects descriptively.
- When two reviews with similar sources of evidence chose to conduct a meta-analysis for the same harm, the resulting estimates were similar in magnitude and direction.
- Standardized hierarchical systems to describe and analyze harms have existed for decades, but we found these systems were not widely used in systematic reviews.
- Reviewers should prespecify and explain their choice of measure(s) to assess harm, including unique harms (e.g., nausea, dizziness), general harm (e.g., “occurrence of any harm”), or surrogates (e.g., drop out due to harm).

## Box. Glossary of terms related to synthesis of harms

**Table:**

Term	Definition
Terms related to harms	
Harms	“Harms”is a general umbrella term to cover the concept of risk that may be associated with an intervention. “Harms”is used to refer to all related ideas, such as adverse events, side effects, tolerability, or safety.
Systematically collected harms	According to The Final Rule, ‘‘‘systematic assessment’ involves the use of a specific method of ascertaining the presence of an adverse event (e.g., the use of checklists, questionnaires, specific laboratory tests at regular intervals)’’. Like a potential benefit of treatment, a systematic AE can be defined using five elements: (11) domain, (2) specific measurement, (3) specific metric, (4) method of aggregation, and (5) time-point [13]. For example, ‘‘proportion of participants with 50% change from baseline to 8 weeks on the Young Mania Rating Scale total score.’’
Non-systematically collected harms	According to The Final Rule, ‘‘‘non-systematic assessment’ relies on the spontaneous reporting of adverse events, such as unprompted self-reporting by participants.’’ Non-systematic adverse events may be collected by asking questions like ‘‘Have you noticed any symptoms since your last examination?’’
Unique harms	A specific harm such as would be reported by someone receiving an intervention, such as “dizziness”, “edema”, or “somnolence”.
General assessment of harm	A non-specific method of assessing harms that aims to summarize multiple aspects of risk into a single measure, such as “occurrence of any harm”, “occurrence of serious adverse events”, or a composite of several unique harms.
Proxy for harm	A surrogate method of assessing harm that is not a direct representation of harm from an intervention, such as “loss-to-follow-up or drop-out due to harms”.
Entry term	The lowest level terms within a hierarchical classification system. Entry terms reflect how an observation might be reported in practice such as “feeling queasy”.
Preferred term	A standardized and distinct descriptor (i.e., single medical concept) for a symptom, sign, disease diagnosis, therapeutic indication, investigation, surgical or medical procedure, and metical social or family history characteristic. Preferred terms have multiple entry terms that may be synonyms or lexical variants of the preferred term, such as “nausea” being the preferred term for the above entry term.
Higher order term	Related preferred terms are grouped together into high level terms based on anatomy, pathology, physiology, aetiology, or function. There are a few hierarchical levels of higher order terms including mid-level body systems (e.g., “Nausea and vomiting symptoms”), upper-level body systems (e.g., “Gastrointestinal signs and symptoms”), and system organ classes (e.g., “Gastrointestinal disorders”) which are groupings by aetiology, manifestation location, or purpose.
COSTART	The Coding Symbols for a Thesaurus of Adverse Reaction Terms is the terminology developed and used by the Food and Drug Administration for the coding, filing, and retrieving of post-marketing adverse reaction reports. It provides a method to deal with the variation in vocabulary used by those submitting adverse event reports to the FDA.
MedDRA	Medical Dictionary for Regulatory Activities is a detailed and highly specific standardized hierarchical medical terminology developed by the International Council for Harmonization of Technical Requirements for Pharmaceuticals for Human Use. MedDRA is designed to facilitate sharing of regulatory information internationally for medical products used by humans and replaced COSTART as the standardized terminology used by the FDA in the late 1990s.
Drug label	A summary for the safe and effective use of a given drug that contains information derived from human experience (both pre and post approval) and is regulated under Code of Federal Regulations Title 21, Subchapter C, Part 201.56.
Indications	The conditions or diseases for which a given drug is used as treatment. Approved indications are those approved by the FDA for marketing and are included on a drug’s label. Physicians can prescribe drugs for indications not approved by the FDA – so called off-label. For example, gabapentin is approved for two on-label indications (postherpetic neuralgia and adjunctive therapy for partial onset seizures) but is commonly prescribed for neuropathic pain among other conditions.
Terms related to review methods	
Pre-specification of harms	An approach to assessing harms in systematic reviews whereby reviewers have one or more harms in mind that they consider important and pre-specify as outcomes of interest for their review. These pre-specified harms are the only harms that are assessed in the review.
No pre-specification of harms (“exploratory”)	An approach to assessing harms in systematic reviews whereby reviewers do not pre-specify any harms of interest as outcomes for their review. Reviewers assess only harms identified in the review process. A review can specify that they will broadly assess harms as an outcome and still be exploratory if they do not note any specific harms of interest.
Descriptive assessment of harm	A narrative description of the harm(s) reported in studies included in the review that does not involve meta-analysis of estimates across studies.
Quantitative assessment of harm	The statistical combination of estimates for harm(s) across two or more studies included in the review. (i.e., meta-analysis for a harm).
Report	Reports are any sources of evidence that are used to provide supporting data in a systematic review. Studies can have multiple public (e.g., journal articles, short reports, registrations, regulatory information) or non-public (e.g., clinical study reports, individual patient data) sources of data (i.e., reports), and these may contain the same or different information about study design features and results.
Corrected Coverage Area	A metric that provides the percentage of overlap in sources of evidence between reviews. Given a set of reviews and their citations, the CCA = (N – r) / ((r x c) – r) where N = total number of citations across the reviews, r = the number of unique citations, and c = the number of reviews in the set.
Selection criteria	The specific rules that are used to define a subset of harms that will be reported among all of the harms collected. Selection criteria are often based on numerical threshold and participant group (e.g., ≥ 5% of participants in the intervention group).
