# Supplementary material for: Harms in Systematic Reviews Paper 3: Given the same data sources, systematic reviews of gabapentin have different results for harms
Source: J Clin Epidemiol. Author manuscript; Available in PMC 2023 Mar 1. (PMC9875741; doi:10.1016/j.jclinepi.2021.10.025)
Supplement: 2 [file NIHMS1858688-supplement-2.docx]

**APPENDIX B – Exploring overlap in sources of evidence for gabapentin harms across conditions**

To further explore the amount of overlap in sources of evidence among reviews, we also examined the overlap in cited reports between conditions (**Figure S**). While the sample of reports contributing to overlap between conditions is small—only 56/514 (11%) unique reports appear in two or more conditions—we can draw three conclusions from the figure:

1. Most reports cited for any given health condition are not cited for other conditions. This is unsurprising because most reviews focused on potential benefits for people with a specific condition.
2. The “condition” with the greatest number of connections to other conditions is “non-specific” (i.e., reviews focused on gabapentin as an intervention and not restricted to a specific condition). This is also unsurprising because these reviews could have reports from any condition.
3. The conditions that share at least one common report—postoperative pain, neuropathic pain, postherpetic neuralgia, fibromyalgia, and migraine—are all pain conditions.

**[Figure S – Network of cited gabapentin reports: shared reports between conditions]**
