# Supplementary material for: Harms in Systematic Reviews Paper 3: Given the same data sources, systematic reviews of gabapentin have different results for harms
Source: J Clin Epidemiol. Author manuscript; Available in PMC 2023 Mar 1. (PMC9875741; doi:10.1016/j.jclinepi.2021.10.025)
Supplement: 3 [file NIHMS1858688-supplement-3.docx]

**APPENDIX C – Gabapentin harms with summary estimates from meta-analyses**

| **Specific harm** | **Measure of effect** | **Estimate (95% CI)** |
| --- | --- | --- |
| “LTFU / Drop-out due to harms” | Number Needed to Harm | 32.5 (18, 22) |
|  | Number Needed to Harm | 26.1 (14.1, 170) |
|  | Odds Ratio | 5.69 (0.25, 128.50) |
|  | Odds Ratio | 1.72 (0.70, 4.22) |
|  | Odds Ratio | 1.57 (0.86, 2.88) |
|  | Odds Ratio | 0.70 (0.25, 1.85) |
|  | Risk Ratio | 3.00 (0.81, 11.15) |
|  | Risk Ratio | 1.99 (1.50, 2.62) |
|  | Risk Ratio | 1.71 (0.71, 4.11) |
|  | Risk Ratio | 1.69 (1.10, 2.60) |
|  | Risk Ratio | 1.4 (1.1, 1.7) |
|  | Risk Ratio | 1.38 (1.14, 1.67) |
|  | Risk Ratio | 0.99 (0.36, 2.74) |
| “Any harms (i.e., non-specific”) | Odds Ratio | 3.48 (0.87, 12.54) |
|  | Odds Ratio | 1.07 (0.91, 1.25) |
|  | Odds Ratio | 0.31 (0.24, 0.41) |
|  | Risk Ratio | 1.66 (1.23, 2.23) |
|  | Risk Ratio | 1.28 (1.22, 1.36) |
|  | Risk Ratio | 1.25 (1.2, 1.3) |
|  | Risk Ratio | 0.91 (0.66, 1.25) |
|  | Risk Difference | 0.05 (-0.04, 0.14) |
|  | Risk | 5.9% |
| “Grouped specific harms” | Odds Ratio | 1.65 (1.25, 2.17) |
|  | Risk Ratio | 1.15 (0.87, 1.51) |
| “Serious harms (i.e., SAEs)” | Risk Ratio | 1.99 (0.23, 17.60) |
|  | Risk Ratio | 1.20 (0.8, 1.7) |
|  | Risk Ratio | 1.19 (0.83, 1.71) |
|  | Risk Ratio | 1.14 (0.6, 2.1) |
|  | Risk Ratio | 1.12 (0.71, 1.77) |
| Dizziness | Number Needed to Harm | 7 (5, 13) |
|  | Odds Ratio | 3.35 (0.32, 35.36) |
|  | Odds Ratio | 3.13 (1.73, 5.66) |
|  | Risk Ratio | 4.45 (2.50, 7.94) |
|  | Risk Ratio | 3.92 (2.55, 6.02) |
|  | Risk Ratio | 3.65 (2.25, 5.92) |
|  | Risk Ratio | 3.16 (1.43, 6.99) |
|  | Risk Ratio | 3.1 (2.6, 3.8) |
|  | Risk Ratio | 2.87 (2.40, 3.44) |
|  | Risk Ratio | 2.71 (1.21, 6.07) |
|  | Risk Ratio | 1.99 (1.17, 3.37) |
|  | Risk Ratio | 1.09 (0.90, 1.32) |
|  | Risk Ratio | 1.06 (0.94, 1.21) |
|  | Risk Ratio | 1.05 (0.95, 1.16) |
|  | Risk Ratio | 1.04 (0.94, 1.15) |
|  | Risk Ratio | 1.02 (0.9, 1.1) |
|  | Risk Ratio | 1.00 (0.88, 1.12) |
|  | Risk Ratio | 0.69 (0.29, 1.60) |
|  | Risk Difference | 0.15 (0.08, 0.22) |
| Vomiting / Nausea | Odds Ratio | 0.92 (0.52, 1.64) |
|  | Odds Ratio | 0.48 (0.23, 0.99) |
|  | Risk Ratio | 2.24 (1.18, 4.24) |
|  | Risk Ratio | 0.90 (0.69, 1.17) |
|  | Risk Ratio | 0.87 (0.60, 1.25) |
|  | Risk Ratio | 0.82 (0.7, 0.9) |
|  | Risk Ratio | 0.81 (0.72, 0.92) |
|  | Risk Ratio | 0.81 (0.72, 0.91) |
|  | Risk Ratio | 0.80 (0.7, 0.9) |
|  | Risk Ratio | 0.79 (0.67, 0.92) |
|  | Risk Ratio | 0.78 (0.69, 0.87) |
|  | Risk Ratio | 0.78 (0.58, 1.04) |
|  | Risk Ratio | 0.77 (0.70, 0.85) |
|  | Risk Ratio | 0.76 (0.67, 0.85) |
|  | Risk Ratio | 0.75 (0.63, 0.89) |
|  | Risk Ratio | 0.67 (0.56, 0.85) |
|  | Risk Ratio | 0.67 (0.59, 0.76) |
|  | Risk Ratio | 0.53 (0.44, 0.63) |
| Somnolence | Number Needed to Harm | 9 (6, 33) |
|  | Odds Ratio | 8.20 (0.40, 169.90) |
|  | Odds Ratio | 2.23 (1.11, 4.46) |
|  | Risk Ratio | 3.92 (2.45, 6.27) |
|  | Risk Ratio | 3.89 (2.22, 6.80) |
|  | Risk Ratio | 3.29 (1.97, 5.48) |
|  | Risk Ratio | 2.9 (2.3, 3.6) |
|  | Risk Ratio | 2.82 (2.27, 3.50) |
|  | Risk Ratio | 2.61 (1.48, 4.62) |
|  | Risk Ratio | 1.50 (1.13, 1.99) |
|  | Risk Ratio | 1.33 (1.0, 1.3) |
|  | Risk Ratio | 1.32 (1.07, 1.65) |
|  | Risk Ratio | 1.22 (1.02, 1.47) |
|  | Risk Ratio | 1.18 (1.09, 1.28) |
|  | Risk Ratio | 1.06 (0.67, 1.67) |
|  | Risk Ratio | 0.91 (0.81, 1.03) |
|  | Risk Difference | 0.11 (0.03, 0.18) |
| Ataxia / Negative myoclonus | Risk Ratio | 17.45 (1.02, 299) |
|  | Risk Ratio | 5.53 (2.49, 12.28) |
|  | Risk Ratio | 4.5 (1.9, 11) |
|  | Risk Ratio | 1.14 (0.80, 1.62) |
| Peripheral edema | Odds Ratio | 8.20 (0.40, 169.40) |
|  | Risk Ratio | 5.09 (1.78, 14.52) |
|  | Risk Ratio | 4.12 (2.66, 6.39) |
|  | Risk Ratio | 3.3 (2.2, 4.9) |
| Fatigue | Odds Ratio | 3.00 (0.51, 17.74) |
|  | Risk Ratio | 2.00 (0.71, 5.69) |
|  | Risk Ratio | 1.85 (1.12, 3.05) |
|  | Risk Difference | -0.03 (-0.08, 0.03) |
| Headache | Odds Ratio | 1.00 (0.06, 17.18) |
|  | Risk Ratio | 1.21 (0.81, 1.82) |
|  | Risk Ratio | 1.17 (0.78, 1.77) |
|  | Risk Ratio | 1.05 (0.82, 1.33) |
| Pruritis | Risk Ratio | 1.24 (0.90, 1.69) |
|  | Risk Ratio | 0.64 (0.51, 0.80) |
|  | Risk Ratio | 0.46 (0.17, 1.26) |
| Visual disturbances | Risk Ratio | 5.72 (1.94, 16.91) |
|  | Risk Ratio | 1.49 (1.14, 1.95) |
|  | Risk Ratio | 1.36 (0.77, 2.40) |
| Mentation / Abnormal thinking | Number Needed to Harm | 20 (11, 100) |
|  | Risk Ratio | 3.34 (1.54, 7.25) |
|  | Risk Difference | 0.05 (0.01, 0.09) |
| Dry mouth | Risk Ratio | 5.97 (0.79, 45.35) |
|  | Risk Ratio | 4.15 (0.54, 31.86) |
|  | Risk Ratio | 1.02 (0.81, 1.29) |
| Pyrexia / Viral infection / Influenza | Number Needed to Harm | 7 (4, 25) |
|  | Risk Difference | 0.03 (-0.03, 0.08) |
|  | Risk Difference | 0.00 (-0.02, 0.03) |
| Weight gain | Risk Ratio | 2.19 (0.86, 5.58) |
|  | Mean increase | 2.23 (0.17, 4.49) |
| Respiratory depression | Risk Ratio | 0.97 (0.45, 2.10) |
|  | Risk Ratio | 0.79 (0.30, 2.10) |
| Insomnia | Risk ratio | 1.22 (0.24, 6.27) |
| Delirium | Risk Ratio | 1.15 (0.87, 1.51) |
| Constipation | Risk Ratio | 0.80 (0.44, 1.44) |
| Confusion | Risk Ratio | 0.50 (0.19, 1.34) |
| Urinary retention | Risk Ratio | 0.64 (0.40, 1.04) |

Cell Colours: “Grey” = Null result

“Red” = Positive statistical association with gabapentin (i.e., GBP harmful)

“Green = Negative statistical association with gabapentin (i.e., GBP protective)
